# Metabolomics and transcriptomics of embryonic livers reveal hypoxia adaptation of Tibetan chickens

**DOI:** 10.1186/s12864-024-10030-w

**Published:** 2024-02-01

**Authors:** Mingming Xue, Runjie Yu, Lixian Yang, Fuyin Xie, Meiying Fang, Qiguo Tang

**Affiliations:** 1 Development Center of Science and Technology, MARA, 100176 Beijing, China; 2 Department of Animal Genetics and Breeding, National Engineering Laboratory for Animal Breeding, MOA Laboratory of Animal Genetics and Breeding, Beijing key Laboratory for Animal Genetic Improvement, College of Animal Science and Technology, State Key Laboratory of Animal Biotech Breeding, Frontiers Science Center for Molecular Design Breeding, China Agricultural University, 100193 Beijing, China

**Keywords:** Tibetan chickens, Metabolomics, Transcriptomics, Embryonic liver, Hypoxia

## Abstract

**Background:**

Exploring the hypoxia adaptation mechanism of Tibetan chicken is of great significance for revealing the survival law of Tibetan chicken and plateau animal husbandry production. To investigate the hypoxia adaptation of Tibetan chickens (TBCs), an integrative metabolomic-transcriptomic analysis of the liver on day 18 of embryonic development was performed. Dwarf laying chickens (DLCs), a lowland breed, were used as a control.

**Results:**

A total of 1,908 metabolites were identified in both TBCs and DLCs. Energy metabolism and amino acid metabolism related differentially regulated metabolites (DRMs) were significantly enriched under hypoxia. Important metabolic pathways including the TCA cycle and arginine and proline metabolism were screened; *PCK1*, *SUCLA2*, and *CPS1* were found to be altered under hypoxic conditions. In addition, integrated analysis suggested potential differences in mitochondrial function, which may play a crucial role in the study of chicken oxygen adaptation.

**Conclusions:**

These results suggest that hypoxia changed the gene expression and metabolic patterns of embryonic liver of TBCs compared to DLCs. Our study provides a basis for uncovering the molecular regulation mechanisms of hypoxia adaptation in TBCs with the potential application of hypoxia adaptation research for other animals living on the Qinghai-Tibet plateau, and may even contribute to the study of diseases caused by hypoxia.

**Supplementary Information:**

The online version contains supplementary material available at 10.1186/s12864-024-10030-w.

## Introduction

The adaptation mechanisms for living at altitude in a hypoxic environment have long been an important scientific issue in the field of evolution and genetics, but they have yet to be fully elucidated. Tissues and organs under hypoxia cannot get enough energy to support basic vital activities, which can lead to problems such as high blood pressure, brain damage, and even death [1, 2]. The liver is one of the most important organs in animals, responsible for biological transformation, metabolism, excretion, regulation, and other physiological functions [3, 4]. Many reports have described that hypoxia can affect liver function, including energy metabolism [5, 6]. Tibetan chickens (*Gallus gallus*; TBCs), an indigenous breed distributed in the Qinghai-Tibet plateau, are a very good model for researching adaptations to hypoxic environments. However, little is known about the metabolic changes due to hypoxia adaptation during embryonic liver development, especially in animals like TBCs that have adapted to the high-altitude environments of the Qinghai-Tibet plateau.

Metabolomics is a powerful tool used to analyze the composition and content changes of small molecule metabolites during a specific period of time. It provides insights into the relative relationship between metabolites and physiological and pathological changes [7]. There are three commonly used detection methods for metabolomics including nuclear magnetic resonance, gas chromatography-mass spectrometry (GC-MS), and liquid chromatograph mass spectrometry (LC-MS) [8]. Tan et al. used GC-MS to perform metabolic analysis on the chicken pectoralis major and serum [9], while Zhang et al. used LC-MS to analyze biomarkers related to the freshness of chilled chicken [10]. Further, Zhang et al. used LC-MS to determine the impact of dietary energy levels on the rumen microbial composition and its relationship to the quality of Black Tibetan sheep meat [11]. However, little metabolomics analysis on the TBC embryonic liver have been reported.

Many studies on the hypoxia adaptation mechanisms of indigenous animals of the Qinghai-Tibet plateau have been carried out using transcriptomics analyses. Related progress has mainly involved energy metabolism, hypoxia response, the Ca^2+^ signaling pathway, and cell survival and proliferation [12, 13]. Zhang et al. have used transcriptomics to analyze the chorioallantoic membrane of TBCs embryos under hypoxia [14]; however, it is necessary to explore the gene expression patterns of the TBC embryonic liver during hypoxia.

This study aimed to better understand the hypoxia adaptation of the TBC embryonic liver by investigating and comparing the fertilized eggs of TBCs and DLCs in normoxia (NTBCs and NDLCs) and simulated high-altitude hypoxic environments (HTBCs and HDLCs). To achieve this, we conduct transcriptome and metabolome analyses on liver tissues collected on day 18 of embryonic development, categorizing them into the HTBC18, HDLC18, NTBC18, and NDLC18 groups. Through this work, we hoped to explore the differences in hypoxia adaptation patterns in the embryonic liver of TBCs and DLCs under different oxygen concentrations and further reveal the potential molecular mechanism of TBC adaptation to hypoxia.

## Results

### Multivariate statistical analysis

**Fig. 1:**
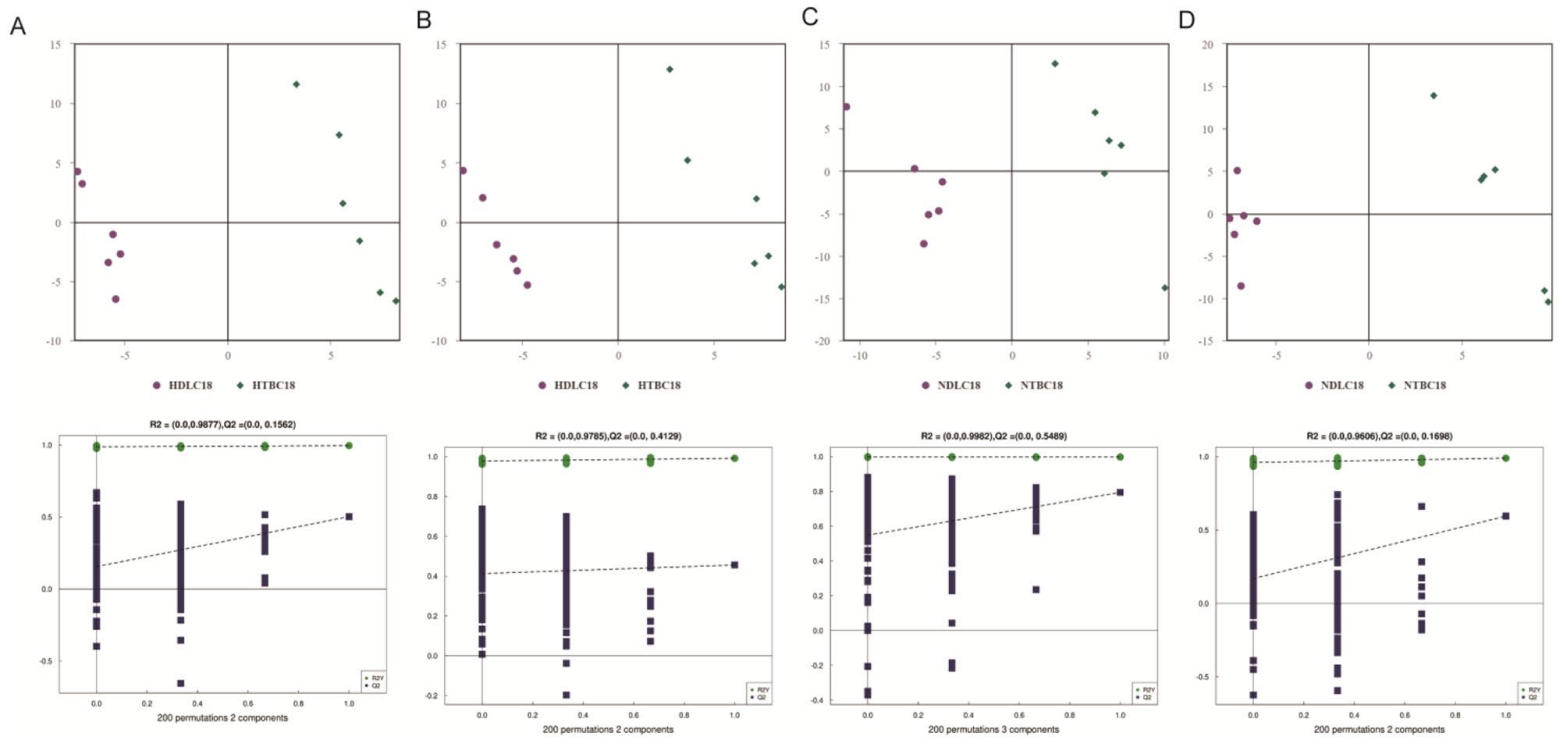
Multivariate analysis of metabolomics in Tibetan chickens (TBCs) and Dwarf laying chickens (DLCs) under normoxia and hypoxia. (**A** and **C**) PLS-DA score plot and permutation test in a positive ion model. (**B** and **D**) Negative ion models in TBCs and DLCs both under hypoxia and normoxia

The principal component analysis (PCA) method was used to observe the overall distribution of metabolites and differences between samples from different groups. The PCA model yielded R2X parameter values of 0.435 in positive ion mode and 0.459 in negative ion mode when comparing HTBC18 and HDLC18 and 0.463 in positive ion mode and 0.489 in negative ion mode when comparing NTBC18 and NDLC18 (Fig. S1). There was an overlap in HTBC18 vs. HDLC18 and NTBC18 vs. NDLC18 comparisons, indicating that each group was not well separated in this model. Partial least-squares discriminant analysis (PLS-DA), a supervised projection method based on the regression extension of PCA, was used to reveal a more apparent segregation of the different groups. The score plot generated by PLS-DA showed a separation between the groups (Fig. 1). The model showed the parameters of Q2 and R2Y were all > 0.5, demonstrating the model fit the data well. Permutation tests further indicated that the model fit the experimental data well (Fig. 1). The score plots from the orthogonal partial least-squares discriminant analysis (OPLS-DA), which was performed to modify the PLS-DA and had enhanced interpretability, also revealed a clear separation of all groups, with good fit and predictability (Fig. S2).

### Metabolite profiles in TBCs and DLCs

**Fig. 2:**
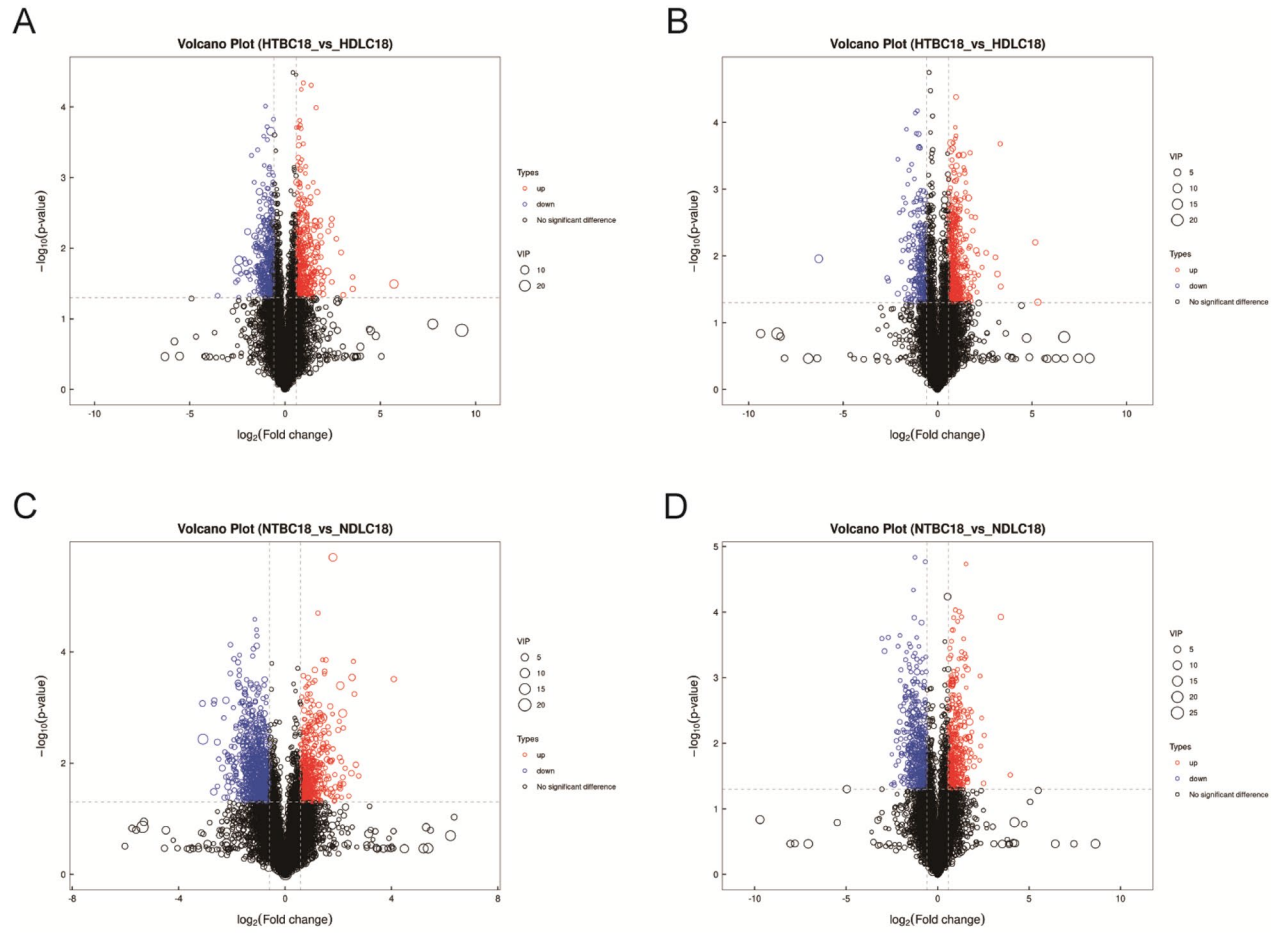
The differentially regulated metabolites (DRMs) by volcano plot in a positive ion model (**A** and **C**) and **a** negitive ion model (**B** and **D**) both under hypoxia and normoxia in TBCs and DLCs. The circles with red and blue are model-separated metabolites with VIP > 1, *P*-value < 0.05, and Foldchange > 1.5 or < 0.67

**Fig. 3:**
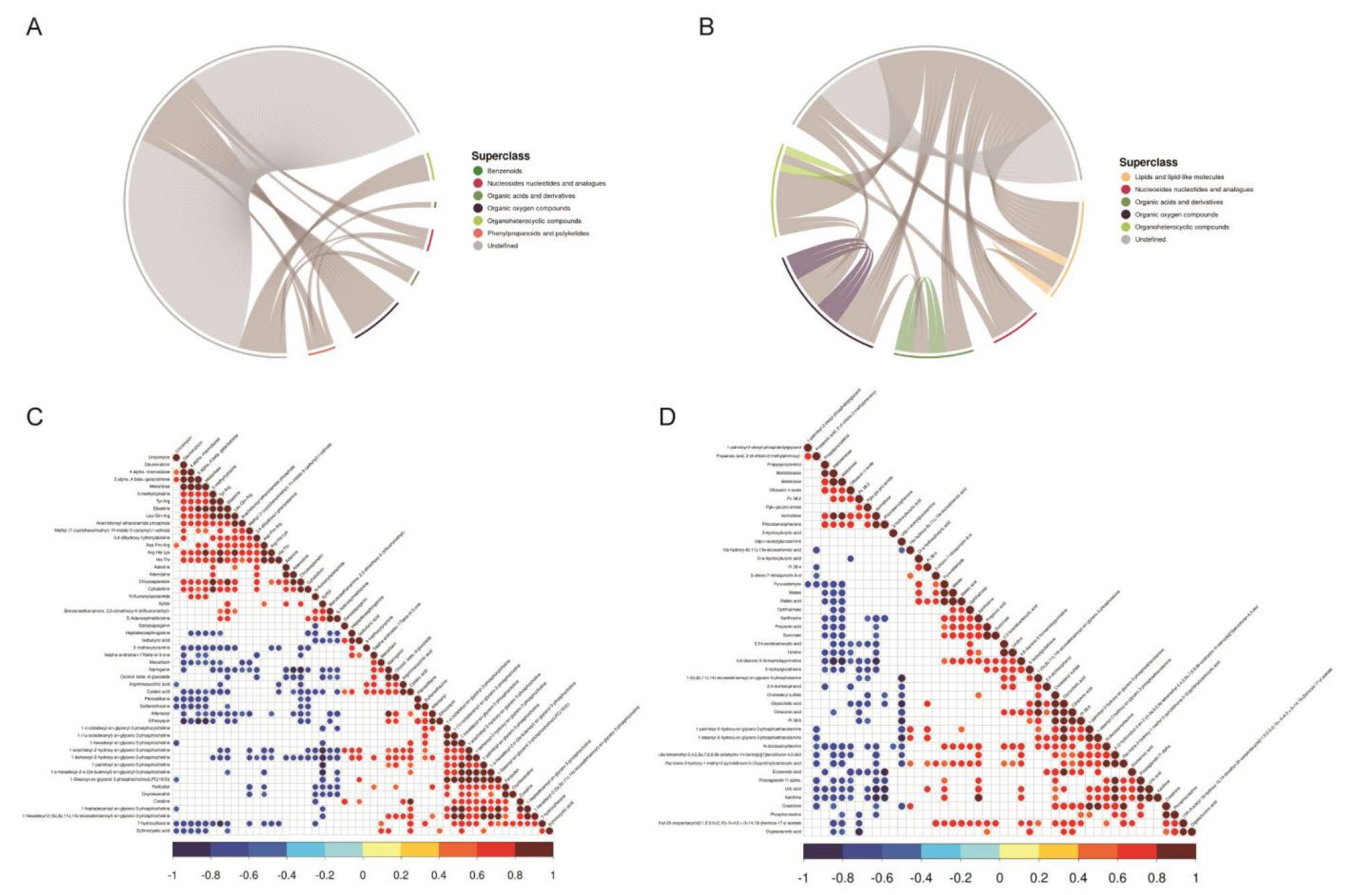
The co-regulatory relationships of DRMs between HTBC18 and HDLC18 groups. (**A** and **B**) The co-regulatory relationships of DRMs in positve and negitive ion models (|r| >0.8 and *P*-value < 0.05). (**C** and **D**) Correlation Heatmaps of DRMs in positive and negitive ion models (VIP > 1, *P*-value < 0.05)

A total of 1,123 metabolites by positive ion mode and 785 metabolites by negative ion mode were initially identified. Figure 2 shows the distribution of differentially regulated metabolites (DRMs) in the different groups (including unidentified). There were 402 and 425 metabolites significantly up-regulated and 490 and 253 metabolites significantly down-regulated in NTBC18 vs. NDLC18 and HTBC18 vs. HDLC18 comparisons, respectively. Using Pearson correlation analysis, we analyzed the correlation between DRMs. Results comparing HTBC18 and HDLC18 groups are shown in Fig. 3. Phenylpropanoids and polyketides had a closer relationship with benzenoids, nucleosides nucleotides and analogues in the positive ion model (Fig. 3A and C), and metabolites were more closely related to each other in the negative ion model (Fig. 3B and D). In addition, the relationship between metabolites was higher in the HTBC18 vs. HDLC18 comparison than in the NTBC18 vs. NDLC18 comparison (Fig. S3). We focused on lipid and oxygen-related components annotated as “lipids and lipid-like molecules” and “organic oxygen compounds” (Table S1 and Table S2). Five differential lipid metabolites were identified only in the HTBC18 vs. HDLC18 comparison including steroid esters ((19r)-9-acetyl-19-hydroxy-10,14-dimethyl-20-oxopentacyclo[11.8.0.0 < 2,10 > 0 < 4,9 > 0.0 < 14,19 >]henicos-17-yl acetate and cholesteryl sulfate), fatty acyls (citraconic acid and eicosenoic acid), glycerophospholipids (phosphatidylcholine (Pc) 36:2). Of these, compared to HDLC18 group, only Pc 36:2 was down-regulated in HTBC18 group. Additionally, one differential or ganic oxygen compound (pyruvaldehyde) was identified between HTBC18 and HDLC18 groups (Table S1).

### Clustering and KEGG analysis of DRMs in TBCs and DLCs

**Fig. 4:**
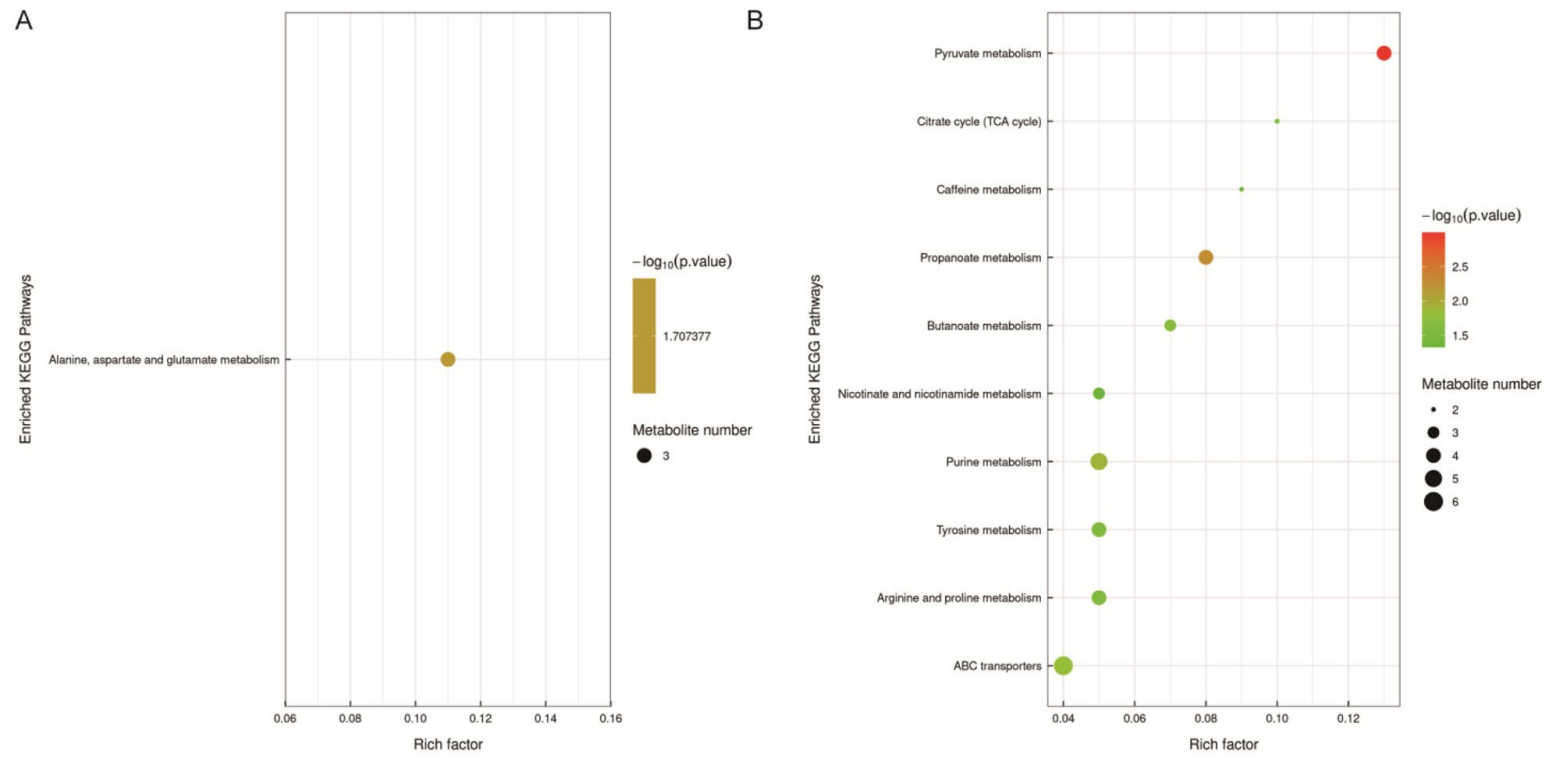
Bubble map of KEGG pathways of DRMs between TBCs and DLCs under normoxia (**A**) and hypoxia (**B**) (*P*-value < 0.05)

To further understand the biological functions of DRMs, we first performed cluster analysis in the NTBC18 vs. NDLC18 and HTBC18 vs. HDLC18 comparisons (Tables S3 and S4). DRMs were enriched by KEGG analysis (Table S5). Significant pathways (*P*-value < 0.05) are shown in Fig. 4. The only significant pathway in the NTBC18 vs. NDLC18 comparison was amino acid metabolism (alanine, aspartate, and glutamate metabolism) (Fig. 4A). DRMs identified in the HTBC18 vs. HDLC18 comparison were significantly enriched in carbohydrate metabolism (pyruvate metabolism, propanoate metabolism, butanoate metabolism, and the citrate cycle (TCA cycle)), nucleotide metabolism (purine metabolism), membrane transport (ATP-binding cassette (ABC) transporters), amino acid metabolism (arginine, proline, and tyrosine metabolism), the biosynthesis of other secondary metabolites (caffeine metabolism), and metabolism of cofactors and vitamins (nicotinate and nicotinamide metabolism) (Fig. 4B). Malate, pyruvaldehyde, succinate, propionic acid, creatine, maleic acid, and 3-methoxytyramine were enriched in carbohydrate and amino acid metabolism, which were all up-regulated except for 3-hydroxybutyric acid and s-adenosylmethionine in HTBC18 groups (Table 1).

### Gene expression profiles in TBCs and DLCs

**Fig. 5:**
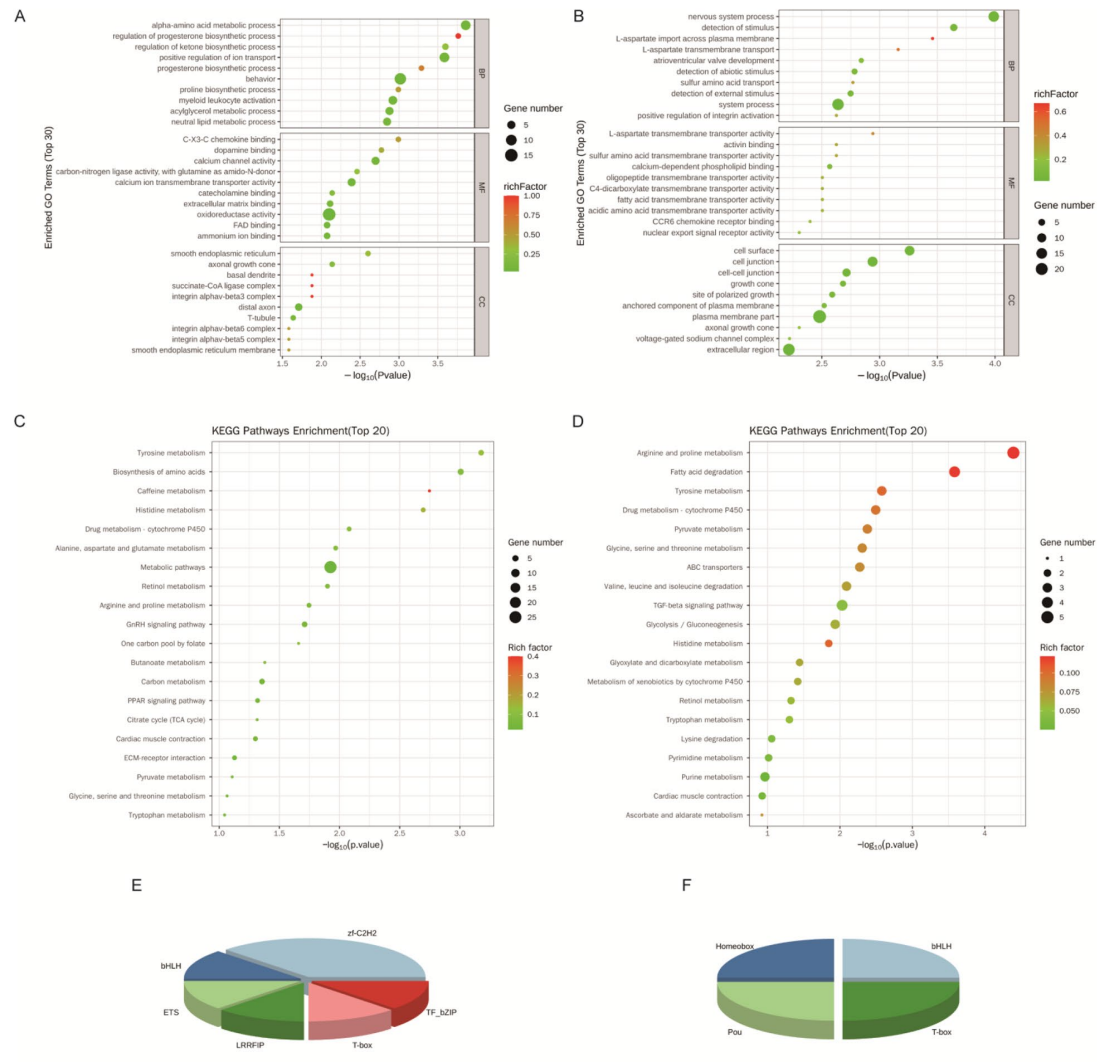
The top 30 differentially expressed genes (DEGs) found by gene ontology (GO) analysis, the top 20 DEGs found by KEGG pathway analysis, and the transcription factor analysis between TBCs and DLCs under normoxia and hypoxia. (**A** and **C**) The top 30 DEGs from GO analysis and the top 20 enriched pathways for DEGs between HTBC18 and HDLC18 groups. (**B** and **D**) The top 30 DEGs from GO analysis and top 20 enriched pathways for DEGs between NTBC18 and NDLC18 groups. Transcription factor analysis between TBCs and DLCs under hypoxia (**E**) and normoxia (**F**). BP: biological process; CC: cellular component; MF: molecular function

We compared the differentially expressed genes (DEGs) in HTBC18 vs. HDLC18 and NTBC18 vs. NDLC18 comparisons. There were 105 and 113 significantly upregulated DEGs and 94 and 121 significantly downregulated DEGs in NTBC18 vs. NDLC18 and HTBC18 vs. HDLC18 comparisons, respectively. Comparing NTBC18 and NDLC18 groups, DEGs were mainly enriched in nervous system process, detection of stimulus, arginine and proline metabolism, and fatty acid degradation (Fig. 5B and D). Comparing HTBC18 and HDLC18 groups, DEGs were mainly enriched in alpha-amino acid metabolic process, regulation of progesterone biosynthetic process, amino acid metabolic pathways, and the TCA cycle (Fig. 5A and C). Transcription factor analysis indicated that zf-C2H2, T-box, bHLH, TF-bZIP, ETS, and LRRFIP were differentially expressed when comparing HTBC18 and HDLC18 (Fig. 5E) and Pou, Homeobox, bHLH, and T-box were differentially expressed when comparing NTBC18 and NDLC18 (Fig. 5F).

**Table 1:** Differentially regulated metabolites (DRMs) between HTBC18 and HDLC18 groups annotated to “carbohydrate metabolism” and “amino acid metabolism” by KEGG enrichment analysis (*P*-value < 0.05)

Pathway	Map ID	Map Name	DRMs
Carbohydrate metabolism	gga00620	Pyruvate metabolism	Malate, Pyruvaldehyde, S-lactoylglutathione, Succinate
	gga00640	Propanoate metabolism	Dl-a-hydroxybutyric acid, Propionic acid, Pyruvaldehyde, Succinate
	gga00020	Citrate cycle (TCA cycle)	Malate, Succinate
	gga00650	Butanoate metabolism	3-hydroxybutyric acid, Maleic acid, Succinate
Amino acid metabolism	gga00330	Arginine and proline metabolism	Creatinine, Phosphocreatine, Creatine, S-adenosylmethionine
	gga00350	Tyrosine metabolism	Maleic acid, Succinate, 3-methoxytyramine, 3,4-dihydroxy-l-phenylalanine

### Integrative metabolomics-transcriptomics analysis in TBCs and DLCs

**Fig. 6:**
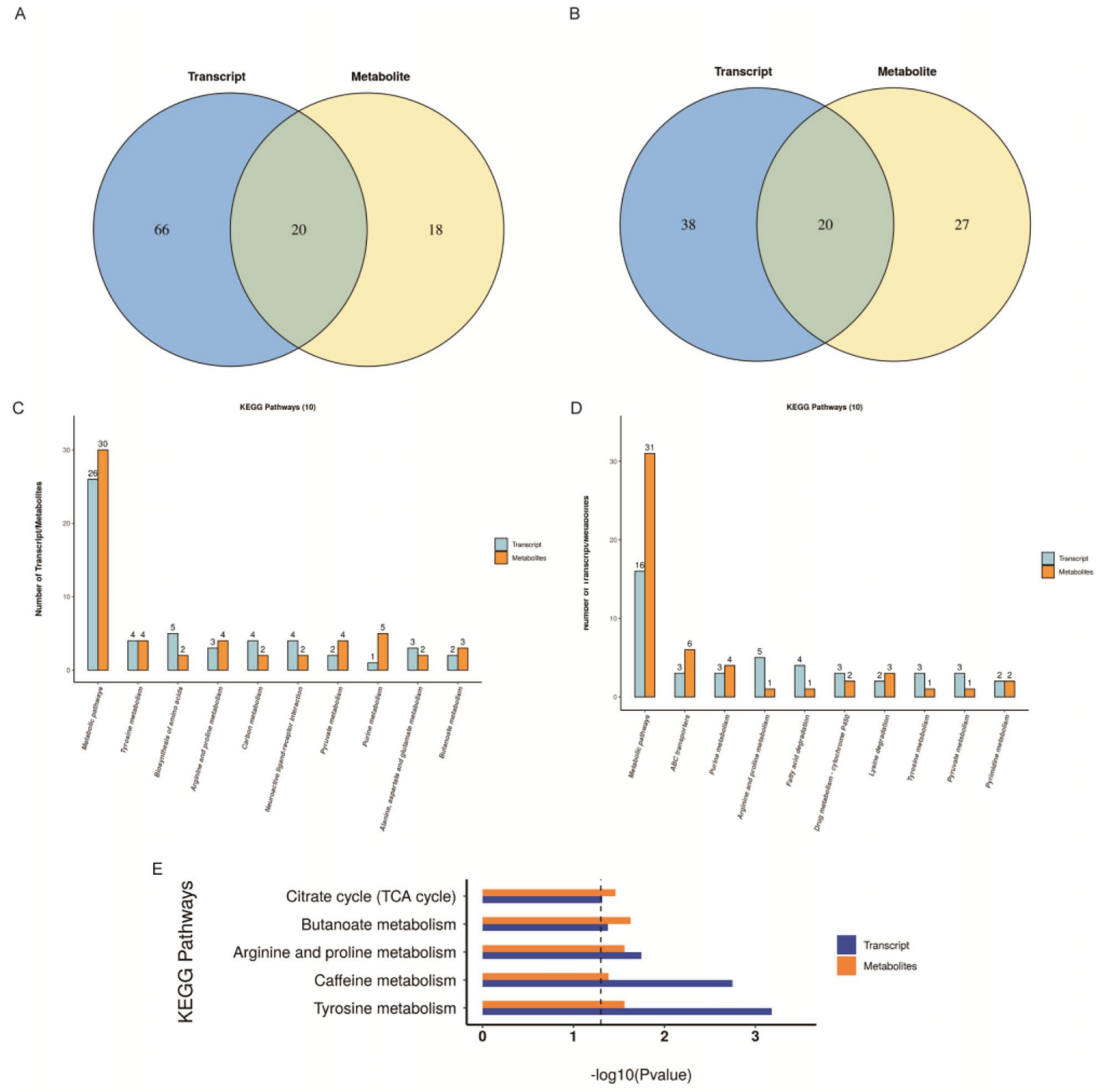
Integrative metabolomic-transcriptomic analysis of TBCs and DLCs under normoxia and hypoxia. Venn diagram of pathways involving differential genes and differential metabolites between HTBC18 and HDLC18 groups (**A**) and between NTBC18 and NDLC18 groups (**B**). The top 10 shared pathways of the largest number of DEGs and DRMs between HTBC18 and HDLC18 groups (**C**) and between NTBC18 and NDLC18 groups (**D**). (**E**) The significantly enriched and shared KEGG pathways of DEGs and DRMs between HTBC18 and HDLC18 groups via KEGG enrichment analysis

To further understand the correlation between DRMs and DEGs, we integrated and analyzed metabolomic and transcriptomic data and the KEGG pathways shared between them. In total, 20 metabolomic and 20 transcriptomic pathways were shared between both NTBC18 and NDLC18 groups and between HTBC18 and HDLC18 groups (Fig. 6A and B). Among the top 10 shared pathways of the largest number of DEGs and DRMs, five pathways were the same between TBCs and DLCs under normoxia or hypoxia, including metabolic pathways, tyrosine, pyruvate, purine metabolism, and arginine and proline metabolism; five pathways were unique to the HTBC18 and HDLC18 comparison group, including biosynthesis of amino acids, carbon metabolism, neuroactive ligand-receptor interaction, butanoate metabolism, alanine, aspartate and glutamate metabolism (Fig. 6C and D). Five pathways including the TCA cycle were significantly enriched between HTBC18 and HDLC18 groups (Fig. 6E). The DEGs between HTBC18 and HDLC18 groups included *PCK1* and *SUCLA2* enriched in the TCA cycle, *ALDH18A1* and *PYCR1* enriched in arginine and proline metabolism, and *CPS1* and *H6PD* enriched in carbon metabolism related to malate and succinate. The DEGs between NTBC18 and NDLC18 groups included ABC transporters *ABCB1* and *ABCC4* and *ALDH9A1*, *ADH1C*, and *ACADSB* enriched in fatty acid degradation related to deoxyinosine and maltotriose.

## Discussion

Low oxygen adaptation to high altitudes has always been a scientific problem attracting wide attention. The environmental oxygen concentration decreases gradually with increased altitude, causing various functional disorders in animals affecting normal growth and development and may even cause death [15]. TBCs deal with this problem by adjusting their physiology [16, 17] and because of this, are a good model to study hypoxic adaptation. Many studies have found that hypoxia adaptation in plateau animals is closely related to metabolism [18–20]. The liver is one of the most important metabolic organs. Metabolic changes in the liver under hypoxia have been well described [21], but no research has been reported how embryonic TBC livers adapt to hypoxia. Metabolomics is a reliable tool for uncovering the relationship between metabolism and phenotype. In this study, we investigated the metabolism patterns of embryonic TBC livers on day 18 of development by integrating analysis of the transcriptome with metabolome data.

Among the NTBC18 and NDLC18 groups, the most diverse DRMs were involved with lipids and lipid-like molecules, organic acids and their derivatives, and organic oxygen compounds. The lipids and lipid-like molecules involved consisted of fatty acyls, glycerophospholipids, prenol lipids, and steroids and steroid derivatives. This result is consistent with previous reports [22, 23]; the liver is important for synthesizing lipids. Through KEGG enrichment analysis, we identified that these enriched metabolites were involved with many pathways, including in amino acid metabolism, membrane transport, cell growth and death, the endocrine system, and signal transduction. The only significantly enriched pathway of amino acid metabolism (alanine, aspartate, and glutamate metabolism) included succinate. Succinate is an intermediate in the TCA cycle, plays a crucial role in mitochondrial ATP production, and acts as a signal for inflammation that stabilizes the transcription factor hypoxia-inducible factor-1a in specific tumors [24, 25]. We found that succinate was up-regulated when comparing HTBC18 and HDLC18 groups. This suggests that succinate may not only be involved in metabolism, but may also play a role in the hypoxia adaptation of TBCs. Furthermore, we observed that D-galacturonic acid and adenosine 5’-monophosphate were associated with membrane transport and signal transduction, highlighting underlying distinctions between TBCs and DLCs under normoxic conditions.

Unlike under normoxia, the most diverse DRMs were mainly enriched in metabolism processes including carbohydrate, amino acid, nucleotide, cofactor, and vitamin metabolism under hypoxia. Carbohydrate metabolism changes included pyruvate metabolism and the TCA cycle. Malate, pyruvaldehyde, s-lactoylglutathione, and succinate were DRMs between HTBC18 and HDLC18; pyruvaldehyde and s-lactoylglutathione were significantly different only under hypoxia. Pyruvaldehyde is the reduction product of pyruvate and s-lactoylglutathione can be used to produce lactate, both of which are important intermediates in pyruvate metabolism [26]. Studies have shown that animals under hypoxia can respond to the challenge by increasing their glycolysis rate to generate energy [27, 28]. Changes in phosphocreatine and creatine were identified as related to amino acid metabolism, including arginine, proline, and tyrosine metabolism. Creatine, the main component of organic acids, is the central component of energy metabolism for resynthesizing ATP [29]. Phosphocreatine reproduces ATP by binding to adenosine diphosphate (ADP) in a reversible reaction catalyzed by creatine kinase [30]. Phosphocreatine is a high-energy molecule capable of resynthesizing ATP much faster than the oxidative phosphorylation and glycolysis processes [31]. These results indicate that TBCs may have higher metabolism and energy supply capacity than DLCs under hypoxia, which is consistent with our previous results under hypoxia conditions in the brain of TBCs and DLCs on day 18 of embryonic development [32]. Differences in membrane transporter (ABC transporters) exist under hypoxia between TBCs and DLCs. The ABC transporter family is one of the largest transporter families and plays an essential function in all living creatures in transporting specific molecules across lipid membranes [33]. The outcomes of our study suggest that hypoxia significantly influences the ABC transporter pathway, highlighting its potential as a pivotal area for further investigation.

We further explored the differential effects of hypoxia on the embryonic liver of TBCs and DLCs by integrating transcriptome and metabolome data. All of pathways were significantly enriched in metabolic pathways by KEGG analysis both under normoxia and hypoxia. We found that hypoxia caused changes in liver metabolism of embryonic TBCs and DLCs. No shared pathways were significant in both omics analyses of NTBC18 and NDLC18 groups, while five shared pathways (TCA cycle, tyrosine metabolism, caffeine metabolism, butanoate metabolism, and arginine and proline metabolism) were significant when comparing HTBC18 and HDLC18 groups. DEGs *PCK1* and *SUCLA2* and metabolites malate and succinate were enrichened and related to the TCA cycle. *PCK1* is the first rate-limiting enzyme in liver gluconeogenesis and catalyzes the conversion of oxaloacetate to phosphoenolpyruvate; hypoxia can upregulate *PCK1* to trigger gluconeogenesis to glycogenolysis [34, 35]. *SUCLA2* encodes the TCA cycle enzyme ADP-specific succinate-CoA ligase (β subunit) and is critical for mitochondrial succinate-CoA ligase and nucleotide diphosphokinase activities [36]. *SUCLA2* can also regulate the succinylation and enzyme activity of glutaminase under oxidative stress, thereby enhancing the level of glutamine metabolism [37].

DEGs *ALDH18A1* and *PYCR1* and metabolites creatine and phosphocreatine enriched in arginine and proline metabolism are of concern. *ALDH18A1* encodes a bifunctional ATP- and NADPH-dependent mitochondrial enzyme which catalyzes the reduction of glutamic acid to delta1-pyrroline-5-carboxylate, a key step in *de novo* synthesis of proline and arginine [38]. *PYCR1* encodes mitochondrial pyrroline 5-carboxylate reductase 1 that catalyzes the NADPH-dependent conversion of pyrroline-5-carboxylate to proline; *PYCR1* activity is increased under hypoxia [39]. In addition, DEG *CPS1*, enriched in carbon metabolism and the biosynthesis of amino acids, also deserves attention. Although no shared pathway was significant in both omics analyses, *CPS1* encodes the mitochondrial enzyme that catalyzes the synthesis of carbamoyl phosphate from ammonia and bicarbonate and also represents a core mitochondrial nucleoid protein [40]. These results suggest that not only amino acid metabolism, but mitochondria should be the focus of our future research under hypoxia.

Among the significantly different pathways, few are noteworthy, such as lipid metabolism. Both TBCs and DLCs had enriched steroid hormone biosynthesis pathways in both normoxia and hypoxia, but the related metabolites were different. Progesterone and 17alpha-hydroxyprogesterone were enriched when comparing NTBC18 and NDLC18 groups, while cholesteryl sulfate and 5alpha-androstan-17beta-ol-3-one were enriched when comparing HTBC18 and HDLC18 groups. 20-Hydroxyarachidonic acid was enriched in the circulatory system of NTBC18 and NDLC18 groups, while adenosine was enriched in the circulatory system of HTBC18 and HDLC18 groups. 20-Hydroxyarachidonic acid is an effective vasoconstrictor and plays a complex role in hypertension, the automatic regulation of cerebral blood flow, and blood-brain barrier (BBB) integrity [41]. Adenosine is a ubiquitous endogenous regulator whose main function is to maintain cell and tissue homeostasis under pathological and stressful conditions and is a potent modulator of inflammation [42]. These results indicate that although similar pathways are enriched under both normoxia and hypoxia, there may be differences in the regulation mode between TBCs and DLCs. The synthesis and degradation of ketone bodies was enriched only under hypoxia. Ketone bodies, a group of fuel molecules that act as an alternative energy source to glucose, are a consequence of lipid metabolism [43]. DEGs *PLA2G4A* and *DGKQ* and metabolite 1-stearoyl-sn-glycerol 3-phosphocholine (LPC (18:0)) were enriched in glycerophospholipid metabolism when comparing HTBC18 and HDLC18 groups and *ALDH9A1*, *ADH1C*, *ACADSB*, and 4-piperidinecarboxamide were enriched in fatty acid degradation when comparing NTBC18 and NDLC18 groups. These results indicate that hypoxia differentially alters lipid metabolism pathways in TBCs and DLCs.

## Conclusions

Through transcriptome and metabolome analysis, we focused on hypoxia adaptation profiling of the embryonic liver under hypoxia on day 18 of development in TBCs and DLCs. In summary, our results showed that in this developmental stage, TBCs and DLCs had different gene and metabolism expression patterns. The main differences were in membrane transport and signal transduction under normoxia and energy metabolism and amino acid metabolism under hypoxia. While under both normoxia and hypoxia lipid metabolism was enriched, the DRMs and metabolic pathways were different. More importantly, vital candidate genes *PCK1*, *SUCLA2*, and *CPS1* and metabolic pathways including the TCA cycle and arginine and proline metabolism were identified that warranted further investigation; subsequent research could focus on mitochondria. These above results provide a basis for uncovering the molecular regulation mechanisms of hypoxia adaptation in TBCs and a potential application of hypoxia adaptation research for other animals living on the Qinghai-Tibet plateau. These results may even contribute to the study of diseases caused by hypoxia.

## Materials and methods

### Sample collection

Fertilized eggs of TBCs and DLCs (100 eggs of each breed per condition) collected at the Experimental Chicken Farm at China Agricultural University (CAU) were transferred to normoxia (21% O_2_) and hypoxia (13% O_2_) incubators and the temperature was maintained at 37.8 °C with a relative humidity of 60% according to our previous article [27]. Day 18 aligns with the major physiological transition during embryonic development, specifically the switch in respiratory patterns [44, 45], making it a key period for studying the impact of oxygen levels on hatchability and survival [46, 47]. Therefore, we focused our investigation on day 18 embryos. Liver tissue was collected on day 18 of embryonic development and frozen in liquid nitrogen immediately for future analysis, as previously described [27].

### RNA extraction and transcriptome analysis

Total RNA was isolated from each liver sample using TRIzol® Reagent following the guidelines provided by the manufacturer (Tiangen, Beijing, China). Subsequently, RNA samples were analyzed based on the A260/A280 absorbance ratio using a Nanodrop ND-2000 system (Thermo Scientific, Waltham, MA, USA). The integrity of RNA was evaluated with an Agilent Bioanalyzer 4150 system (Agilent Technologies, Santa Clara, CA, USA).

Paired-end libraries were prepared using an ABclonal mRNA-seq Lib Prep Kit (Abclonal, Wuhan, China) following the manufacturer’s instructions. The library preparations were sequenced on an Illumina Novaseq 6000 (Illumina, San Diego, CA, USA) and 150 bp paired-end reads were generated. Raw data (raw reads) of the fastq format were processed through in-house perl scripts removing reads containing adapter sequences, reads containing ploy-N, and low-quality reads from the raw data. The reference genome (ftp://ftp.ensembl.org/pub/release-108/fasta/gallus_gallus/dna/) and gene model annotation files (ftp://ftp.ensembl.org/pub/release-108/gtf/gallus_gallus/) were downloaded from genome websites directly. The reference genome underwent indexing before aligning the paired-end clean reads using Hisat2 v2.0.5 [48]. Subsequently, FeatureCounts v1.5.0-p3 was employed to enumerate the reads mapped to individual genes [49]. Calculation of the Fragments Per Kilobase of transcript per Million mapped reads (FPKM) for each gene was performed based on the gene length and the corresponding mapped read counts. Differential expression analysis was performed using the DESeq2 R package (1.16.1). Genes with Padj < 0.05 and|log_2_ fold change| >1 were considered to be differentially expressed [50].

### Enrichment and transcription factor analysis

The clusterProfiler R software package was used for Gene Ontology (GO) function enrichment and Kyoto Encyclopedia of Genes and Genomes (KEGG) pathway enrichment analyses [51]. For the transcription factor analysis, we annotated DEGs using the AnimalTFDB database (http://bioinfo.life.hust.edu.cn/AnimalTFDB/). Differentially expressed transcription factors were categorized based on their transcription factor families.

### Metabolite extractions and LC-MS/MS analysis

Liver tissues were cut on dry ice (~ 80 mg) into 2 mL Eppendorf tubes and were homogenized with 200 μL of H_2_O and five ceramic beads. The homogenized solution underwent metabolite extraction by adding 800 μL of a methanol/acetonitrile mixture (1:1, v/v). Following centrifugation for 20 min at 14,000 g and 4 °C, the resulting supernatant was dried using a vacuum centrifuge. Subsequently, for LC-MS analysis, the dried samples were reconstituted in 100 μL of an acetonitrile/water solvent (1:1, v/v) and then centrifuged at 14,000 g for 15 min at 4 °C. The resultant supernatant was used for injection [52].

LC-MS/MS analysis was previously described in the published work [53]. In brief, the analysis was performed using a UHPLC (Vanquish UHPLC, Thermo Fisher Scientific, Waltham, MA, USA) coupled to an Orbitrap (Shanghai Applied Protein Technology Co., Ltd, Shanghai, China). Hydrophilic interaction liquid chromatography separation was conducted using a 2.1 mm x 100 mm ACQUIY UPLC BEH Amide 1.7 μm column (Waters, Wexford, Ireland). Subsequently, sample solution was aerosolized in both positive and negative modes of electrospray ionization (ESI). Mass spectrometry (MS) data were analyzed using the freely available XCMS software. The collection of Algorithms of MEtabolite pRofile Annotation was employed for annotation isotopes and adducts. Only variables with more than 50% of their nonzero measurement values in at least one group were retained from the extracted ion features. Metabolite compound identification was executed by comparing the accuracy of the m/z value.

### Statistical analysis

Statistical significance was assessed using one-way analysis of variance (ANOVA) to test homogeneity of variances via Levene’s test, followed by a Student’s t-test. Prism 7.0 (GraphPad Software Inc., San Diego, CA, USA) was employed for calculations and figure plotting. Differences were considered to be statistically significant for *P*-values < 0.05. Scale bars represented the standard error of the mean (SEM) from at least three separate experiments.

Following sum-normalization, the processed data were analyzed by R package (ropls), as previously described [54]. This involved multivariate data analysis, including Pareto-scaled PCA and OPLS-DA. Then, the robustness of the model was evaluated through 7-fold cross-validation and response permutation tests. The variable importance in the projection (VIP) value of each variable in the OPLS-DA model was calculated to indicate its contribution to the classification. A Student’ s t test was applied to determine the significance of differences between two groups of independent samples. Variables with VIP > 1 and *P*-value < 0.05 were considered to screen for significantly changed metabolites. Pearson’s correlation analysis was performed to determine the correlation between two variables.

### Electronic supplementary material

Below is the link to the electronic supplementary material.

**Additional file 1: Supplementary Figure S1.** PCA score plots in TBCs and DLCs. **Supplementary Figure S2.** Multivariate analysis of metabolomics in TBCs and DLCs. **Supplementary Figure S3.** Co-regulatory relationships of DRMs between NTBC18 and NDLC18 groups

**Additional file 2: Supplementary Table S1.** DRMs annotated to “lipids and lipid-like molecules” and “organic oxygen compounds” in human metabolome database (HMDB) between HTBC18 and HDLC18 groups

**Additional file 3: Supplementary Table S2.** DRMs annotated to “lipids and lipid-like molecules” and “organic oxygen compounds” in HMDB between NTBC18 and NDLC18 groups

**Additional file 4: Supplementary Table S3.** The DRMs in positive and negative ion models between HTBC18 and HDLC18 groups

**Additional file 5: Supplementary Table S4.** The DRMs in positive and negative ion models between NTBC18 and NDLC18 groups

**Additional file 6: Supplementary Table S5.** KEGG Enrichment analysis of DRMs in NTBC18_vs_NDLC18 groups and HTBC18_vs_HDLC18 groups

## Data Availability

The data presented in this study are available on request from the corresponding authors.
