# Supplementary material for: Metabolomics and transcriptomics of embryonic livers reveal hypoxia adaptation of Tibetan chickens
Source: BMC Genomics. 2024 Feb 1;25:131. doi: 10.1186/s12864-024-10030-w (PMC10832288; doi:10.1186/s12864-024-10030-w)
Supplement: Supplementary file 1 — Additional file 1: Supplementary Figure S1. PCA score plots in TBCs and DLCs. Supplementary Figure S2. Multivariate analysis of metabolomics in TBCs and DLCs. Supplementary Figure S3. Co-regulatory relationships of DRMs between NTBC18 and NDLC18 groups [file 12864_2024_10030_MOESM1_ESM.docx]

**Supplementary materials**


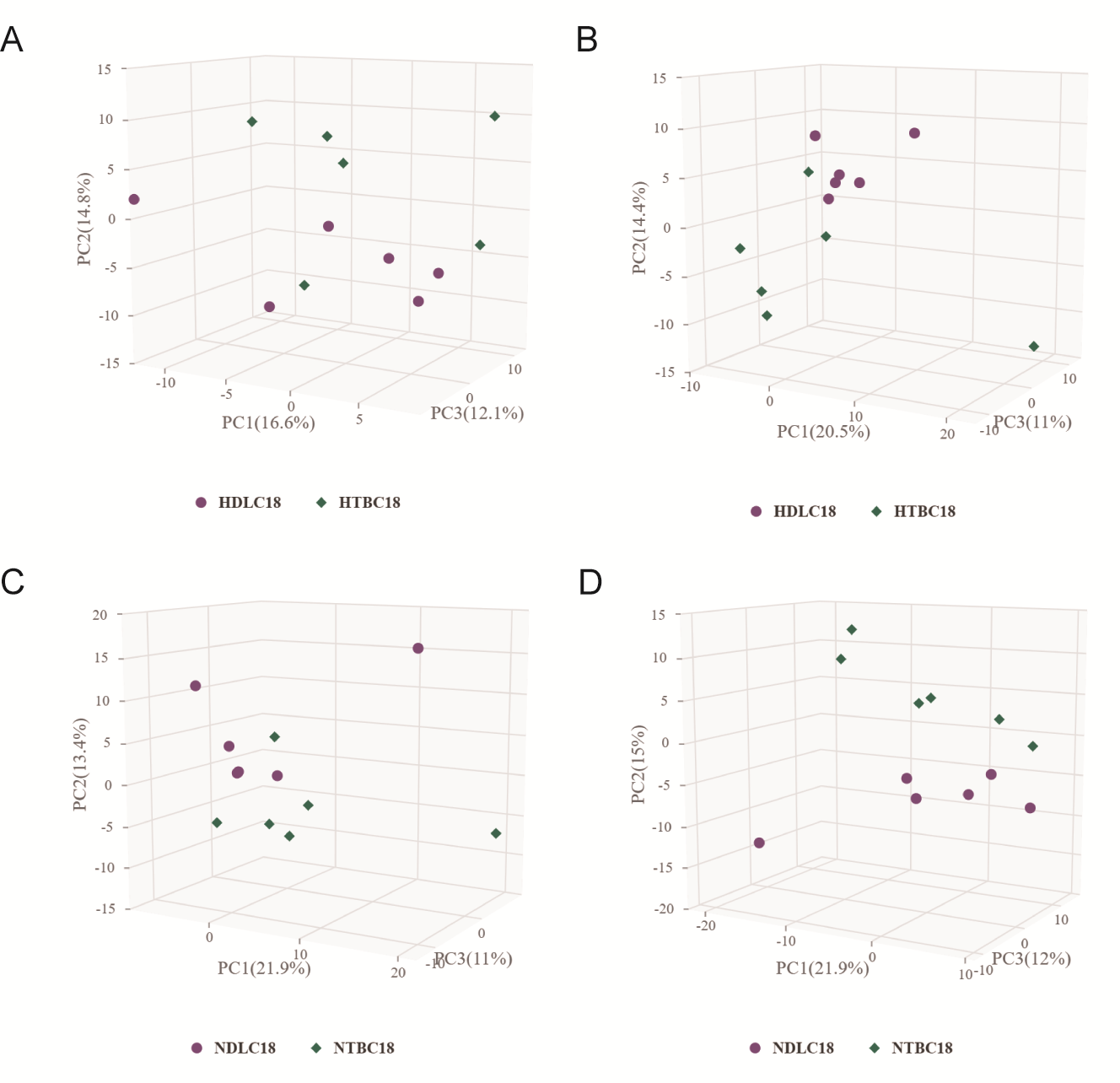


**Supplementary Figure S1. PCA score plots in TBCs and DLCs under normoxia and hypoxia.** PCA score plot in positive (A) and negative (B) ion models under hypoxia; PCA score plot in positive (C) and negative (D) ion models under normoxia.


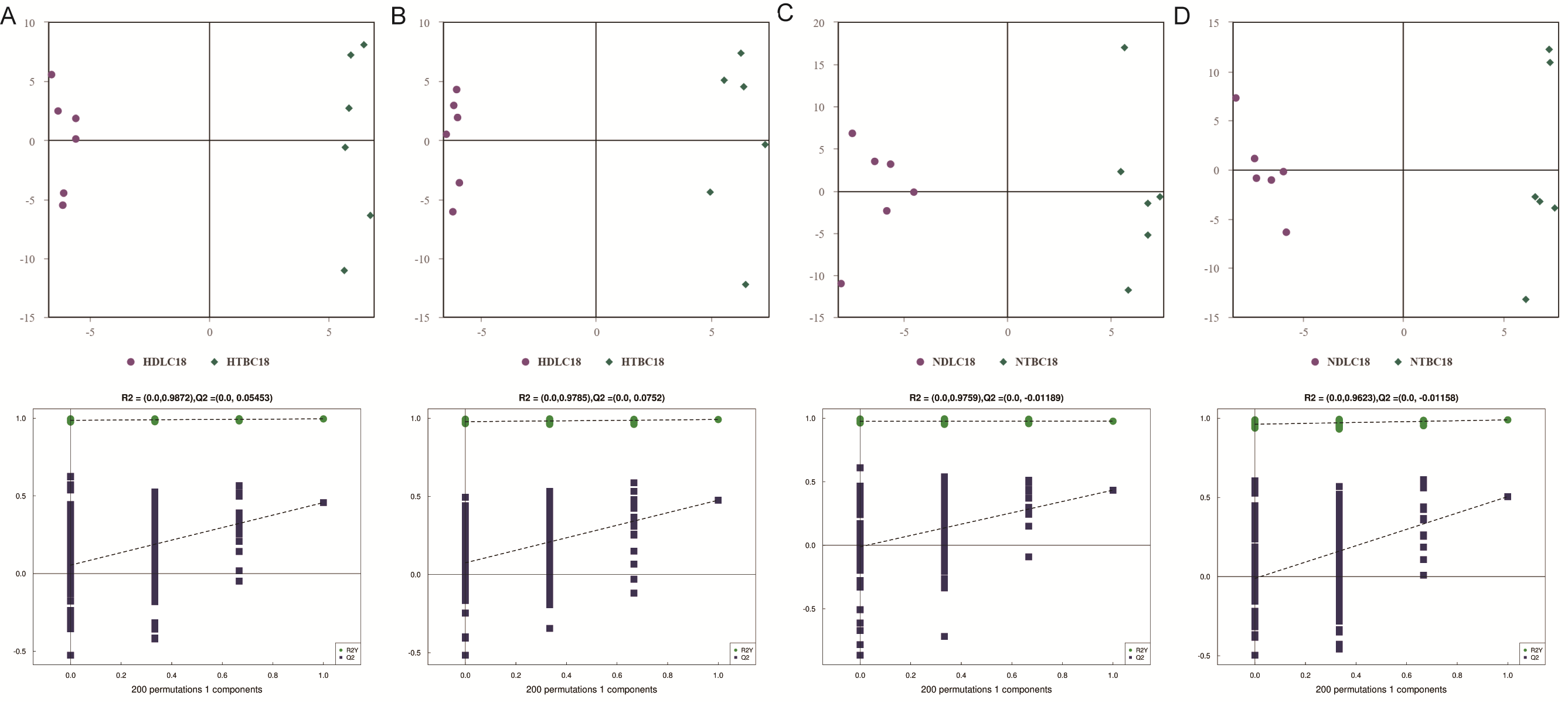


**Supplementary Figure S2.** **Multivariate analysis of metabolomics in TBCs and DLCs under normoxia and hypoxia.** (A and C) OPLS-DA score plot and permutation test in a positive ion model. (B and D) Negative ion models in TBCs and DLCs both under hypoxia and normoxia.


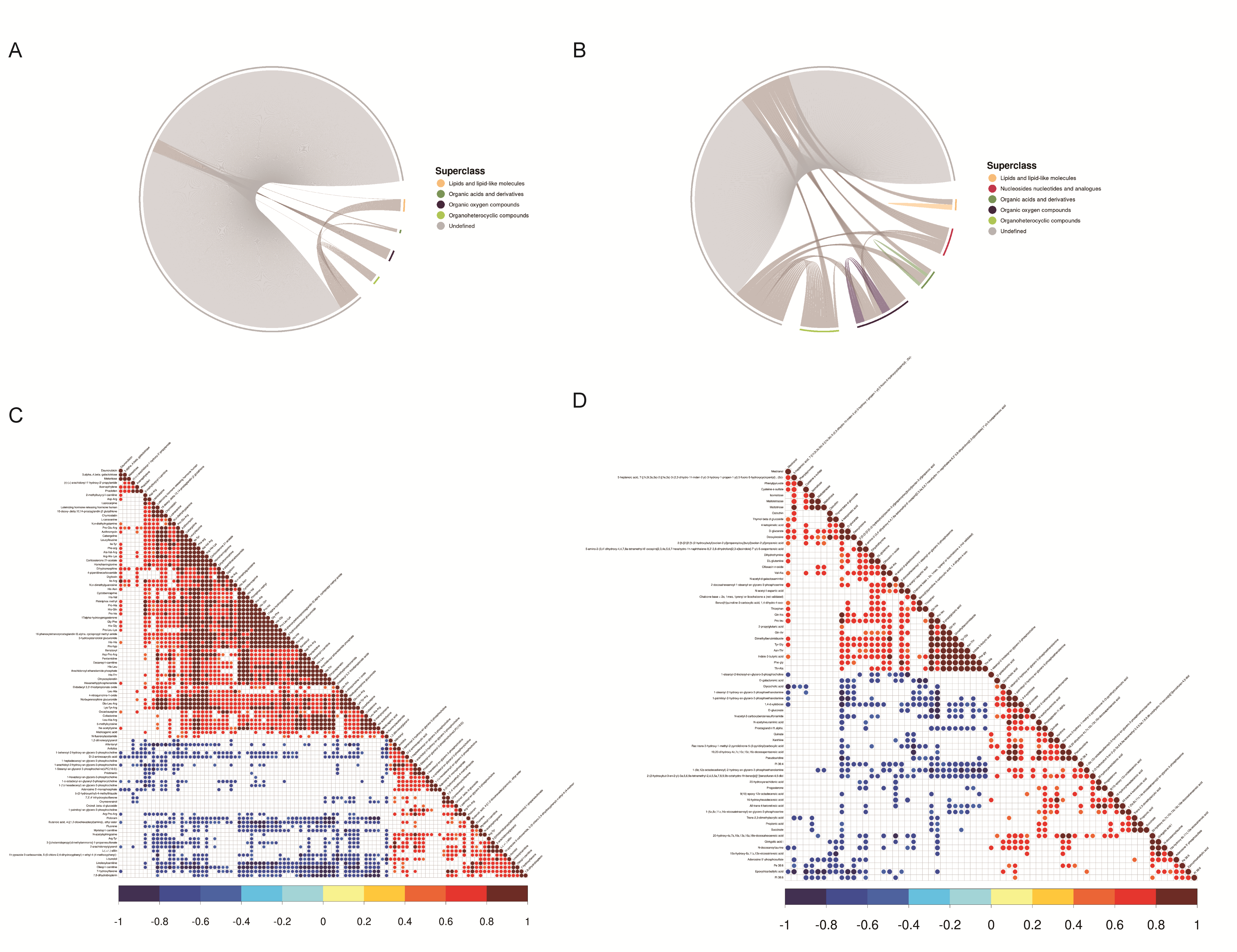


**Supplementary Figure S3.** **Co-regulatory relationships of DRMs between NTBC18 and NDLC18 groups under normoxia.** (A and B) The co-regulatory relationships of DRMs in positive and negative ion models (|r| >0.8 and *P*-value <0.05); (C and D) Correlation Heatmap of DRMs in positive and negative ion models (|r| >0.8 and *P*-value <0.05).
