# Supplementary material for: Metabolomics and transcriptomics of embryonic livers reveal hypoxia adaptation of Tibetan chickens
Source: BMC Genomics. 2024 Feb 1;25:131. doi: 10.1186/s12864-024-10030-w (PMC10832288; doi:10.1186/s12864-024-10030-w)
Supplement: Supplementary file 2 — Additional file 2: Supplementary Table S1. DRMs annotated to “lipids and lipid-like molecules” and “organic oxygen compounds” in human metabolome database (HMDB) between HTBC18 and HDLC18 groups [file 12864_2024_10030_MOESM2_ESM.docx]

**Supplementary Table S1.** DRMs annotated to “lipids and lipid-like molecules” and “organic oxygen compounds” in human metabolome database (HMDB) between HTBC18 and HDLC18 groups.

| Compounds | SuperClass (HMDB) | RT (min) | m/z |
| --- | --- | --- | --- |
| (19r)-9-acetyl-19-hydroxy-10,14-dimethyl-20-oxopentacyclo[11.8.0.0<2,10>.0<4,9 >.0<14,19>]henicos-17-yl acetate | Lipids and lipid-like molecules | 20.520500 | 503.28373 |
| 1-palmitoyl-2-hydroxy-sn-glycero-3-phosphoethanolamine | Lipids and lipid-like molecules | 257.523000 | 452.27830 |
| 1-palmitoyl-2-oleoyl-phosphatidylglycerol | Lipids and lipid-like molecules | 208.769000 | 747.51639 |
| 1-stearoyl-2-hydroxy-sn-glycero-3-phosphoethanolamine | Lipids and lipid-like molecules | 254.071000 | 480.30935 |
| 15s-hydroxy-8z,11z,13e-eicosatrienoic acid | Lipids and lipid-like molecules | 47.048000 | 321.24364 |
| Cholesteryl sulfate | Lipids and lipid-like molecules | 23.842450 | 465.30408 |
| Citraconic acid | Lipids and lipid-like molecules | 507.263000 | 129.01930 |
| Eicosenoic acid | Lipids and lipid-like molecules | 39.564700 | 309.28024 |
| Glycocholic acid | Lipids and lipid-like molecules | 246.499000 | 464.31440 |
| Isomaltose | Lipids and lipid-like molecules | 437.821000 | 221.06669 |
| N-docosanoyltaurine | Lipids and lipid-like molecules | 36.947600 | 446.33078 |
| Pc 36:2 | Lipids and lipid-like molecules | 212.093000 | 844.60660 |
| Pi 36:4 | Lipids and lipid-like molecules | 246.341000 | 857.51831 |
| Pi 38:6 | Lipids and lipid-like molecules | 245.506000 | 881.51837 |
| Prostaglandin f1.alpha. | Lipids and lipid-like molecules | 133.633000 | 337.23820 |
| Maltotetraose | Organic  oxygen compounds | 495.176000 | 383.11932 |
| Maltotriose | Organic  oxygen compounds | 495.060000 | 503.16167 |
| Pyruvaldehyde | Organic  oxygen compounds | 420.725000 | 71.01385 |
