# Supplementary material for: Metabolomics and transcriptomics of embryonic livers reveal hypoxia adaptation of Tibetan chickens
Source: BMC Genomics. 2024 Feb 1;25:131. doi: 10.1186/s12864-024-10030-w (PMC10832288; doi:10.1186/s12864-024-10030-w)
Supplement: Supplementary file 3 — Additional file 3: Supplementary Table S2. DRMs annotated to “lipids and lipid-like molecules” and “organic oxygen compounds” in HMDB between NTBC18 and NDLC18 groups [file 12864_2024_10030_MOESM3_ESM.docx]

**Supplementary Table S2.** DRMs annotated to “lipids and lipid-like molecules” and “organic oxygen compounds” in HMDB between NTBC18 and NDLC18 groups.

| Compounds | SuperClass(HMDB) | RT(min) | m/z |
| --- | --- | --- | --- |
| 1-(9z,12z-octadecadienoyl)-2-hydroxy-sn-glycero-3-phosphoethanolamine | Lipids and lipid-like molecules | 253.711000 | 476.27816 |
| 1-palmitoyl-2-hydroxy-sn-glycero-3-phosphoethanolamine | Lipids and lipid-like molecules | 257.523000 | 452.27830 |
| 1-stearoyl-2-hydroxy-sn-glycero-3-phosphoethanolamine | Lipids and lipid-like molecules | 254.071000 | 480.30935 |
| 1-stearoyl-2-linoleoyl-sn-glycero-3-phosphocholine | Lipids and lipid-like molecules | 216.847000 | 820.56269 |
| 15s-hydroxy-8z,11z,13e-eicosatrienoic acid | Lipids and lipid-like molecules | 47.048000 | 321.24364 |
| 16-hydroxyhexadecanoic acid | Lipids and lipid-like molecules | 58.692100 | 271.22794 |
| 19,20-dihydroxy-4z,7z,10z,13z,16z-docosapentaenoic acid | Lipids and lipid-like molecules | 68.860900 | 361.23816 |
| 2-docosahexaenoyl-1-stearoyl-sn-glycero-3-phosphoserine | Lipids and lipid-like molecules | 243.539000 | 834.52896 |
| 2-propylglutaric acid | Lipids and lipid-like molecules | 357.223000 | 173.09316 |
| 20-hydroxy-4z,7z,10z,13z,16z,18e-docosahexaenoic acid | Lipids and lipid-like molecules | 48.781600 | 343.22775 |
| 20-hydroxyarachidonic acid | Lipids and lipid-like molecules | 57.635550 | 319.22797 |
| 5-heptenoic acid, 7-[(1r,2r,3s,5s)-2-[(1e,3s)-3-(2,3-dihydro-1h-inden-2-yl)-3-hydroxy-1-propen-1-yl]-3-fluoro-5-hydroxycyclopentyl]-, (5z)- | Lipids and lipid-like molecules | 23.420300 | 381.23144 |
| 9(10)-epoxy-12z-octadecenoic acid | Lipids and lipid-like molecules | 52.743700 | 295.22792 |
| All-trans-4-ketoretinoic acid | Lipids and lipid-like molecules | 59.566550 | 269.21232 |
| Glycocholic acid | Lipids and lipid-like molecules | 246.499000 | 464.31440 |
| Isomaltose | Lipids and lipid-like molecules | 437.821000 | 221.06669 |
| Mestranol | Lipids and lipid-like molecules | 23.169900 | 309.17415 |
| N-docosanoyltaurine | Lipids and lipid-like molecules | 36.947600 | 446.33078 |
| Ostruthin | Lipids and lipid-like molecules | 28.573800 | 297.15302 |
| Pe 38:6 | Lipids and lipid-like molecules | 209.420000 | 762.50758 |
| Pi 36:4 | Lipids and lipid-like molecules | 246.341000 | 857.51831 |
| Pi 38:6 | Lipids and lipid-like molecules | 245.506000 | 881.51837 |
| Progesterone | Lipids and lipid-like molecules | 107.703000 | 313.23859 |
| Prostaglandin f1.alpha. | Lipids and lipid-like molecules | 133.633000 | 337.23820 |
| Thymol-beta-d-glucoside | Lipids and lipid-like molecules | 27.842100 | 311.16872 |
| Trans-2,3-dimethylacrylic acid | Lipids and lipid-like molecules | 431.583000 | 99.00777 |
| 1,4-d-xylobiose | Organic oxygen compounds | 331.149000 | 341.12538 |
| D-galacturonic acid | Organic oxygen compounds | 355.216000 | 113.02445 |
| D-glucarate | Organic oxygen compounds | 275.341000 | 209.02378 |
| D-gluconate | Organic oxygen compounds | 437.944000 | 195.05098 |
| Maltotetraose | Organic oxygen compounds | 495.176000 | 383.11932 |
| Maltotriose | Organic oxygen compounds | 495.060000 | 503.16167 |
| N-acetyl-d-galactosaminitol | Organic oxygen compounds | 316.274000 | 222.09829 |
| N-acetylneuraminic acid | Organic oxygen compounds | 421.142000 | 308.09877 |
| Quinate | Organic oxygen compounds | 388.720000 | 191.05616 |
